# Patch variability following carotid endarterectomy: a survey of Great Britain and Ireland

**DOI:** 10.1308/003588412X13373405385494

**Published:** 2012-09

**Authors:** GJ Harrison, JA Brennan, JB Naik, SR Vallabhaneni, RK Fisher

**Affiliations:** Royal Liverpool and Broadgreen University Hospitals NHS Trust,UK

**Keywords:** Carotid endarterectomy, Variability, Technique, Patch angioplasty, Primary closure

## Abstract

**INTRODUCTION:**

Evidence suggests a clinical benefit with patch angioplasty after carotid endarterectomy (CEA). The UK National Vascular Database has demonstrated variation in practice but does not record technical details. This study was intended to define indications and technique of patching after CEA.

**METHODS:**

An electronic questionnaire was emailed to all 402 members of the Vascular Society of Great Britain and Ireland. The email could not be received by 23 and 14 did not perform CEA. Some questions allowed multiple answers. Fisher’s exact test was used for statistical analysis.

**RESULTS:**

There were 187 responses (51%). Fifteen members (8%) performed eversion CEA, which obviates patching. Of all the respondents, 121 surgeons (65%) always use a patch. Seventy of these (58%) use the full patch width (median: 8mm, range: 4–10mm). Fourteen (12%) variably trimmed the patch (median: 7.5mm, range: 5–10mm) and 34 (28%) routinely trimmed (median: 6mm, range: 3–20mm). Selective patching, dependent on internal carotid artery diameter, was performed by 48 respondents (26%), 23 of whom specified a median artery threshold diameter of 5mm (range: 3–8mm). General anaesthesia was always or usually used by 83 surgeons (45%), local anaesthesia by 77 (41%) and the remainder followed patient choice. Obligatory patching is performed by 68 of the 83 respondents (82%) who prefer general anaesthesia whereas only 40 of the 77 surgeons (52%) who use local anaesthesia always patch (*p*<0.0001).

**CONCLUSIONS:**

There is a variable rate of patching after CEA in the UK, which appears dependent on the vessel size and mode of anaesthesia. There are also differences in the patch width adopted.

Carotid endarterectomy (CEA) is the standard treatment for significant, symptomatic carotid artery stenosis.^1,2^ Evidence for prompt surgical treatment^3^ has resulted in recommendations for surgery to be within two weeks of symptoms.^4^ These guidelines, however, make few recommendations regarding operative techniques, which remain controversial.

A recent randomised trial demonstrated equivalent outcomes between local and general anaesthesia.^5^ There is also the option of standard or eversion endarterectomy, with the latter avoiding the use of a patch. Controversies in the standard CEA include the use of a shunt, tacking sutures and closure of the arteriotomy with or without a patch angioplasty.^6^ When a patch is adopted, materials include autologous vein, biological and synthetic patches.^7^

Vascular surgeons in the UK are strongly recommended to enter data into the National Vascular Database to record peri-operative parameters and outcomes.^8^ Information on the use of carotid patches and type of anaesthesia is recorded. However, there is no detailed information on the indications for patch angioplasty, nor the patch material or size. Experimental evidence has suggested that patch width affects geometry and haemodynamics following surgery, which may influence clinical outcomes.^9,10^ The aim of this questionnaire-based study was to further explore the indications and techniques of patching after CEA adopted by vascular surgeons across Great Britain and Ireland.

## Methods

**Figure 1:**
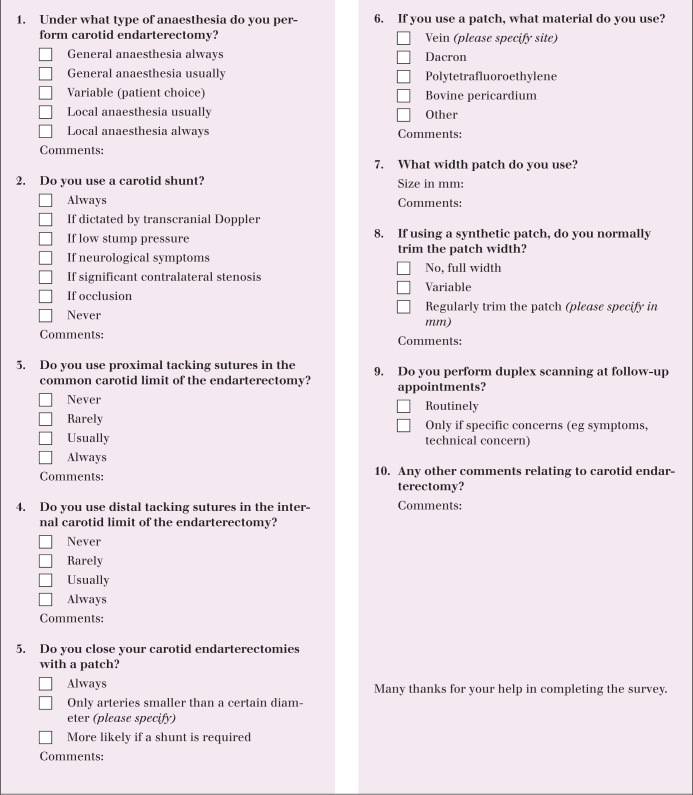
Questionnaire sent to members of the Vascular Society

An electronic survey (Fig 1) was composed by a panel of five vascular surgeons exploring the use of patches, different anaesthetic modalities and indications for intra-operative shunting. In addition, there was a question regarding post-operative duplex surveillance. Most questions were multiple choice, with a free text box available for comments.

The email addresses of all ordinary members of the Vascular Society of Great Britain and Ireland were obtained from the 2010 handbook. Questionnaires were sent to all listed vascular consultants. Surgical trainees and interventional radiologists were excluded as they would not perform CEA independently. Recruitment for the study was closed four weeks after the last email had been sent and responses were anonymous. Multiple answers were possible and some respondents did not answer all questions. If an answer was given as a range, the midpoint was taken for subsequent analysis. Statistical analysis was performed using Quick-Calcs (GraphPad Software, La Jolla, CA, US) using the two-tailed Fisher’s exact test with *p*<0.05 set as the threshold for significance.

## Results

The questionnaire was emailed to the 402 eligible members of the Vascular Society. The email could not be received by 23 members and 14 responded to confirm that they did not perform CEA. In total, 187 surgeons responded out of the potential 365 (51%).

Patch angioplasty following CEA was practised routinely by 121 members (65%). Selective patching, dependent on internal carotid artery diameter, was adopted by a further 48 surgeons (26%). Two additional respondents (1%) were more likely to patch when using a shunt. Fifteen members (8%) performed eversion CEA as their primary technique, thereby obviating a patch, and one member (0.5%) never patched.

Of the 121 surgeons who always used a patch, 70 used the full width of the patch (median: 8mm, range: 4–10mm), 34 routinely trimmed (median: 6mm, range: 3–20mm, with the exception of one response the maximum was 10mm) and 14 variably trimmed the patch (median: 7.5mm, range: 5–10mm). Of the 48 surgeons who indicated selective use of a patch based on arterial diameter, 18 (37%) used the full size patch, 18 (37%) normally trimmed the patch and 10 (21%) variably trimmed the patch. (Two respondents did not specify.) The indication for a patch was specified as a median internal carotid artery diameter of 5mm (range: 3–8mm) by 23 surgeons, a female patient by 7 surgeons and the inability to insert a Pruitt shunt by 1 surgeon.

Dacron was used by 115 surgeons (59%) (median width: 7mm, range: 2–20mm) while bovine pericardium was used by 46 respondents (24%) (median width: 8mm, range: 3–10mm). Seventeen (9%) used polytetrafluoroethylene (PTFE) patches and six (3%) used polyurethane patches. Seven surgeons (4%) used vein patches; two specified the great saphenous vein from the groin, one the great saphenous vein from the ankle and one the external jugular vein.

General anaesthesia was always used by 48 surgeons (26%), with a further 35 surgeons (19%) stating a preference for general anaesthesia. CEA was always performed under local anaesthesia by 28 members (15%) and another 49 members (26%) preferred local anaesthesia. Patient choice was followed by 27 surgeons (14%).

An intra-operative shunt was used in all cases by 64 surgeons (34%), selectively by 120 surgeons (65%) and never by 2 surgeons (1%). The indications for selective shunting included the presence of neurological symptoms for 98 members (82%), the use of general anaesthesia for 20 (17%), stump pressure measurements for 19 (16%), transcranial Doppler for 17 (14%) and the presence of a contralateral carotid occlusion for 16 (13%). The use of a shunt and a patch by the 83 surgeons regularly performing CEA under general anaesthesia and the 77 surgeons predominantly using local anaesthesia are summarised in Table 1.

**Table 1:** Obligatory patch and shunt use by modality of anaesthesia

	Number of respondents	Shunt always	Patch always
General anaesthesia	83	58 (70%)	68 (82%)
Local anaesthesia	77	2 (3%)	40 (52%)
Significance		*p*<0.0001	*p*<0.0001

Ninety-four surgeons (51%) never use common carotid artery tacking sutures and 77 (42%) use them rarely. Eight (4%) usually tack the common carotid artery and six (3%) always do. Four (2%) never tack the internal carotid artery, 78 (43%) rarely tack, 79 (44%) usually tack and 22 (12%) always tack. Routine post-operative duplex surveillance is performed by 65 surgeons (35%) and 119 (65%) stated that duplex was reserved for specific concerns such as symptoms. Of the 121 members who routinely patch, 84 (70%) selectively perform duplex at follow-up appointments, compared with 27 of 47 (57%) who primarily close or selectively patch (*p*=0.151).

## Discussion

CEA is one of the most frequently performed vascular operations, with over 6,000 performed annually in the UK.^8^ The benefit of surgery is a reduction in future stroke risk. However, this is dependent on a low incidence of peri-operative stroke and death. It is therefore imperative that surgical complications are minimised. Despite this, there remains controversy over several technical aspects of CEA. This study has demonstrated the wide variation in the indications for patch angioplasty, according to surgeons’ preference, other factors that influence patch use and the resulting differences in patch dimension used.

In this survey, 26% of surgeons patch selectively, dictated primarily by internal carotid artery diameter. Rerkasem and Rothwell performed a meta-analysis of 10 trials involving 1,967 patients undergoing 2,157 operations, comparing patch closure with primary closure.^6^ This suggested a reduction in rate of peri-operative ipsilateral stroke with patching (odds ratio: 0.31, 95% confidence interval: 0.15–0.63, *p*=0.001). However, the authors commented that the strength of this finding was limited by numerous weaknesses including the quality of trials, which were generally considered to be poor, the small numbers of participants, and variability of inclusion criteria and outcome measures. Exclusion criteria in some of the trials were an internal carotid artery diameter of <3.5mm ^11^ to 5mm.^12,13^ This range is comparable with that in our study, which identified a median diameter of <5mm as being an indication to patch for a number of surgeons.

The risk of post-operative complications may be increased in such small diameter vessels and one may anticipate a greater benefit afforded by patch angioplasty. The exclusion of such patients, likely to be predominantly female, may therefore confound the results. Indeed, one study in the meta-analysis showed female patients to be at a significantly higher risk of restenosis.^14^ Our study found that female sex was considered an indication for selective patching by a proportion of surgeons.

The choice of anaesthesia during CEA remains controversial despite the large, multicentre GALA trial, which showed no significant outcome difference between local and general anaesthesia.^5^ However, between the two groups there was a difference in the use of patches: 42% of patients under local anaesthesia compared with 50% under general anaesthesia (*p*<0.001). The benefit with patching suggested by Rerkasem and Rothwell^6^ may explain the failure to demonstrate improved outcomes with local anaesthesia. The findings in our study corroborate the higher rate of patch use (82% vs 52% obligatory) with general anaesthesia. It may be that with local anaesthesia the operation is performed more hurriedly as patching may add approximately 15 minutes to the length of the operation.

Our study demonstrates wide variation in the size of patch used, with median widths in unmodified patches of 8mm, in variably trimmed patches of 7.5mm and in routinely adapted patches of 6mm. These differences will affect the geometry of the reconstructed vessel. The effect of adding a patch to an artery will add to its circumference and, consequently, the radius. Increasing the radius will decrease the wall shear stress, which is the stress applied parallel or tangential to the arterial wall and related to the flow rate, blood viscosity and decreasing arterial radius. Low wall shear stress has been shown to promote neointimal hyperplasia and restenosis may follow.^15–17^

Experimental evidence comparing repair of a canine common carotid artery with primary closure, a 5mm patch or a 10mm patch demonstrated different geometry and more disturbed haemodynamics with the larger patch.^9^ This may cause flow recirculation and platelet aggregation,^18^ which may result in peri-operative embolic events and occlusion as well as contributing to late restenosis through neointimal hyperplasia. These detrimental influences on the haemodynamics with the 10mm patch reconstruction would appear to be in contradiction to the clinical evidence of the benefit with patching. Primary closure has been shown to reduce arterial dimensions.^9^ The inevitable result of this would be a residual stenosis. Patching may also reduce the possibility of technical errors. There remains little research in this field and detailed in vivo and bench studies are required to further elucidate the haemodynamic effect of a patch.

A range of different patch materials is available including vein, dacron, PTFE and bovine pericardium, with differing benefits and disadvantages. Synthetic patches have the advantage of being readily available but with an inherent infection risk.^19^ Our survey demonstrated a six-fold preference for dacron over PTFE. A further meta-analysis by Rerkasem and Rothwell**compared different patch materials.^7^ This did not support the use of vein over synthetic patches in reduction of peri-operative stroke (OR: 1.22, 95% CI: 0.70–2.13) but there was limited evidence to support the use of PTFE over dacron (long-term stroke OR: 10.58, 95% CI: 1.34–83.43). Bovine pericardium benefits from being easy to handle and has minimal suture line bleeding.^20^ Bovine patch infection has been reported^21^ but the risk is thought to be lower as it is biological. Bovine pericardium is preferred by 24% of surgeons despite having a limited evidence base.^22^

The limitations of this study include the relatively low response rate (51%) and the subjective nature of the data. However, it does offer the most comprehensive information on current practice and opinion in Great Britain and Ireland. All Vascular Society members are recommended to submit data to the UK CEA audit, contained in the National Vascular Database.^8,23^ The submitted data overlap with some of the areas in our study, with concurrence between the outcomes from our study and ‘round 1’ (December 2005 – December 2007) of the UK CEA audit (Table 2). This would suggest that the data derived from our questionnaire are accurate, thereby validating our findings.

**Table 2:** Operative techniques recorded by the National Vascular Database (NVD) and our surgeon survey

Parameter	NVD	Survey
Eversion carotid endarterectomy	6%	8%
Shunt insertion	43%	46% always
Patch angioplasty closure	68%	65% always

A survey sent to GALA trial participants showed much greater use of routine patch angioplasty and shunting in the UK than in the rest of Europe.^24^ The key additional information from our survey is the variation in patch trimming and the resulting patch size used. Furthermore, our respondents were not limited to those participating in a clinical trial and may better reflect actual practice.

## Conclusions

Our study objectively demonstrates variations in the use and technique of patch angioplasty following CEA, according to surgeons’ preference, with approximately a quarter of Vascular Society members remaining unconvinced by current evidence and guidelines. The variation in patch size also indicates an area of controversy.
